# B-cell imaging with zirconium-89 labelled rituximab PET-CT at baseline is associated with therapeutic response 24 weeks after initiation of rituximab treatment in rheumatoid arthritis patients

**DOI:** 10.1186/s13075-016-1166-z

**Published:** 2016-11-18

**Authors:** Stefan Bruijnen, Michel Tsang-A-Sjoe, Hennie Raterman, Tamara Ramwadhdoebe, Daniëlle Vugts, Guus van Dongen, Marc Huisman, Otto Hoekstra, Paul-Peter Tak, Alexandre Voskuyl, Conny van der Laken

**Affiliations:** 1 Amsterdam Rheumatology and immunology Center (ARC), location VU University Medical Center, Amsterdam, The Netherlands; 2 Amsterdam Rheumatology and immunology Center (ARC), location Academic Medical Center, University of Amsterdam, Amsterdam, The Netherlands; 3 Department of Radiology & Nuclear Medicine, VU University Medical Center, Amsterdam, The Netherlands

**Keywords:** Rheumatoid arthritis, Positron-emission tomography, Rituximab, Clinical response, Treatment monitoring

## Abstract

**Background:**

B cells are key players in the pathogenesis of rheumatoid arthritis (RA). Although successful in 50–60% of patients with RA, anti-B-cell therapy given as rituximab could be more efficient by identifying potential responders prior to treatment. Positron emission tomography (PET) using radiolabeled rituximab for B-cell imaging might provide the means to fulfil this unmet clinical need. The objective of this study was to investigate the association between biodistribution of zirconium-89 (^89^Zr)-rituximab on PET-computed tomography (CT) and clinical response in patients with RA.

**Methods:**

We included 20 patients with RA who were starting rituximab treatment. At the first intravenous (i.v.) therapeutic dose, patients were also injected with ^89^Zr-rituximab, followed by PET-CT. European League Against Rheumatism (EULAR) response criteria were applied to determine response at week 24. PET-CT was analyzed visually and quantitatively. Lymph node (LN) biopsies were performed at 0 and 4 weeks to correlate B-cell counts with imaging data.

**Results:**

PET-positive hand joints (range 1–20) were observed in 18/20 patients. Responders had significantly higher ^89^Zr-rituximab uptake in PET-positive hand joints than non-responders (median target-to-background (T/B)) ratios (IQR) were 6.2 (4.0–8.8) vs. 3.1 (2.2–3.9), *p* = 0.02). At T/B ≥4.0, positive and negative predictive values for clinical response were respectively 90% and 75%. Quantitative ^89^Zr-rituximab hand joint uptake on PET correlated inversely with CD22^+^ B-cell count in LN tissue at 4 weeks of treatment (*r* = 0.6, *p* = 0.05). In addition, the CD22^+^ B-cell count in LN correlated positively with quantitative LN PET data at baseline, supporting the specificity of B-cell imaging on PET.

**Conclusions:**

Non-invasive B-cell imaging by ^89^Zr-rituximab PET-CT has promising clinical value to select RA responders to rituximab at baseline. ^89^Zr-rituximab PET-CT may also hold promise for monitoring anti-B-cell therapies in other B-cell driven autoimmune diseases, such as systemic lupus erythematosus and Sjögren’s disease.

**Electronic supplementary material:**

The online version of this article (doi:10.1186/s13075-016-1166-z) contains supplementary material, which is available to authorized users.

## Background

B cells play an important role in the pathophysiological process of rheumatoid arthritis (RA), presumably through B-T-cell interaction and auto-antibody production. Targeted depletion of B cells with a monoclonal antibody (mAb) such as rituximab (anti-CD20) appears to be efficient and cost-effective in patients with RA that is refractory to disease-modifying anti-rheumatic drugs (DMARDS) and anti-tumor necrosis factor-alpha therapy (anti-TNF) [1–3]. Nevertheless, 30–50% of patients with RA do not respond to rituximab [4, 5]. Treatment could be more efficient if potential responders to rituximab could be selected before treatment or early during treatment.

Molecular imaging with positron emission tomography (PET) might be a predictive tool for therapeutic outcome in RA: PET allows non-invasive 3D visualization and quantification of pathophysiological processes at the picomolecular level, by binding of radiolabeled agents to any affected tissue in the whole body [6]. Apart from prediction of disease outcome in RA [7, 8], our group has also previously demonstrated that PET predicts infliximab outcome as early as two weeks after initiation of treatment [9]. This predictive value for therapeutic outcome was later confirmed by Roivainen et al. for early DMARD combination treatment [10].

In our laboratory we have experience with good manufacturing practice (GMP)-labeling of mAbs with the PET isotope Zirconium-89 (^89^Zr) [11, 12]. Zirconium-89 has a physical half-life of about 78.4 hours and can be stably coupled to mAbs. ^89^Zr-labeled rituximab has been successfully applied for imaging and radioimmunotherapy of CD20-positive B-cell lymphomas [13]. In fact, this “immuno-PET” technique showed more tumor-positive lymph nodes than the standard fluorodeoxyglucose (^18^F-FDG; glucose metabolism) PET scans [13]. ^89^Zr-rituximab PET imaging may not only be interesting for visualization of B cells in B-cell lymphoma but also for other B-cell-related immune activity in the body. In RA, apart from the joint synovium, studies have demonstrated that B cells also play an important role in lymph nodes in patients with RA [14–16].

## Methods

In this study, we investigated whether in vivo biodistribution of ^89^Zr-rituximab in RA, with special focus on hand joints and lymph nodes, was associated with clinical response to rituximab. We also collected lymph node biopsies for analysis of B cells prior to rituximab treatment, and after treatment with rituximab for 4 weeks, in order to investigate the potential association between histological findings and imaging results.

### Patients

Twenty rituximab-naïve patients with RA were included between October 2010 and November 2014. Inclusion criteria were: patients (>18 years of age) with at least two clinically inflamed joints in the hands/wrists and a clinical indication for rituximab treatment, and stable treatment with DMARDs for at least 2 weeks and previous failure or intolerance to at least one anti-TNF drug. Anti-TNF had to be discontinued at least 4 weeks before initiation of rituximab treatment. Patients were not eligible if being treated with >10 mg daily dose of prednisolone at the time of inclusion, if they had been treated with investigational drugs within the previous 3 months or if they were pregnant or breast-feeding. The study protocol was approved by the VUmc Medical Ethics Committee. All patients gave written informed consent prior to participation in the study.

## Study design

### Rituximab treatment and administration of ^89^Zr-rituximab

**Fig. 1:**
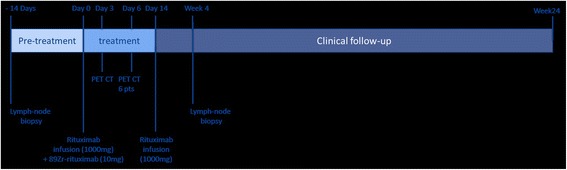
Schematic overview of the study design. *PET* positron emission tomography, *CT* computer tomography

The overall design of the study is shown in Fig. 1. In concordance with routine clinical practice, patients received two times 1000 mg rituximab at day 0 and at day 14, respectively. Premedication with intravenous (i.v.) methylprednisolone was omitted to study the specific effects of rituximab on disease activity. Within one hour, the first rituximab infusion was followed by infusion of 10 mg rituximab labeled with 18 mega Becquerel (MBq) ^89^Zr. The infusion system was flushed twice with 20 ml NaCl 0.9% and residual activity in the administration device was measured afterwards. Blood samples were taken respectively at 5 minutes, 60 minutes and 72 hours and (in a subpopulation) 144 hours post injection (p.i.), to determine ^89^Zr-rituximab kinetics based on measurements of radioactivity in blood and plasma.

### Synthesis of ^89^Zr-rituximab

We obtained ^89^Zr (2.7 GBq/mL in 1 M oxalic acid) from Perkin Elmer (Boston, MA, USA). The ^89^Zr-Rituximab was produced in a current Good Manufacturing Practice (cGMP) compliant way in a facility with a manufacturing license at the university campus (Amsterdam, The Netherlands) essentially the same as described before [13, 17].

### PET-CT scanning

PET-CT scans were performed three days after tracer administration due to long residence time of intact mAbs combined with the half-life of zirconium-89 of 78.4 hours [11] (see Additional file 1: Appendix A). In addition, scans were performed 6 days p.i. in a subpopulation of six patients to investigate targeting of ^89^Zr-rituximab over time in relation to blood clearance. In short, whole body and detailed scans of the hands/wrists were obtained. The maximum total scan time was approximately 60–75 minutes per patient. All scans were reconstructed according to international guidelines [18].

### PET imaging analysis

Biodistribution and extra-articular uptake (e.g. lymph nodes) as depicted with whole body PET was qualitatively interpreted by one experienced nuclear medicine physician (OSH). Detailed images of the wrists/hands were subsequently interpreted by two independent readers (OSH and SBR) for PET positivity (dichotomous) of metacarpophalangeal (MCP), proximal interphalangeal (PIP) and wrist joints (*n* = 22 in total per patient), with an adjudicator (JvdL) in case of discrepancies. Visual PET positivity was defined as clearly enhanced tracer uptake in joints versus local background. All readers were blinded to the clinical data.

For quantitative analysis, volumes of interest (VOIs) were drawn using analyzing software developed in house [18] with the low-dose CT as the anatomical reference. Specific details are described in Additional file 1: Appendix A. Essentially, to analyze ^89^Zr-rituximab biodistribution in the body, VOIs were drawn on the aortic arch (representing blood pool activity), lymph nodes, bone marrow, large joints and internal organs, using in-house biodistribution standard operating procedures. For the comparison of quantitative joint uptake in the wrist/hands at PET with clinical follow up, VOIs were drawn on top of the PET-positive joints. In addition, the metacarpal bone was used as background to calculate local target-to-background (T/B) ratios. To compare quantitative uptake of visually PET-negative joints we applied fixed-size VOIs. Such VOIs were drawn on the wrists, MCP joints and PIP joints, centered in the middle of the joint. Standardized uptake values (SUV) were calculated, defined as radioactivity in the VOI normalized for injected dose and patient weight.

### Lymph node biopsy

Up to 2 weeks before and 4 weeks after initiation of rituximab treatment, lymph node biopsies were performed as described previously [19]. In short, an accessible lymph node in the inguinal region was selected by ultrasound examination. In arthritis of the knee or ankle, joint lymph node biopsies were performed on the ipsilateral side. After the incision, needle biopsies were obtained using a semi-automated biopsy gun and six to eight biopsies were obtained and processed immediately for immunohistochemical (IHC) analysis.

### IHC analysis of lymphoid tissue sections

For detailed information see Additional file 2: Appendix B. Basically, for IHC analysis, the lymph node tissue sections were stained using mouse mAbs against B cells (CD22), T cells (CD3), and plasma cells (CD138). CD22 was used as the Bcell marker instead of CD20 because of the potential blockade of CD20 receptors during rituximab treatment. Staining was analyzed by digital image analysis as described previously [20] and the number of positive cells was calculated as the number of positive cells per square millimetre of stained lymphoid tissue section.

### Clinical follow up

Clinical follow up was performed at 24 weeks. Data collected included disease activity score of 28 joints (DAS28), erythrocyte sedimentation rate (ESR), C-reactive protein (CRP) and Health Assessment Questionnaire (HAQ) at baseline and week 24. A joint was defined clinically active if tenderness and/or swelling were present on clinical examination. All clinical data were obtained by an experienced research nurse blinded to the imaging data. Response to rituximab treatment was defined based on the response criteria of the European League Against Rheumatism (EULAR) at 24 weeks [21], and in this study moderate responders were considered as responders. All treating physicians were blinded to the imaging data.

## Statistics

Data obtained by visual observation was analyzed in a descriptive manner. Data are presented as mean ± standard deviation (SD) or as median and interquartile range (IQR) in case of skewed distribution. The paired-sample Wilcoxon signed-rank test, the Mann–Whitney *U* test or Fisher’s exact test were used as appropriate. Correlation between PET and clinical outcome parameters was assessed using Spearman rank tests. A *p* value <0.05 was regarded as statistically significant. Statistical analyses were performed using SPSS version 20.0.0 for Windows (SPSS, Chicago, IL, USA).

## Results

### Clinical data

PET-CT scans were obtained in 20 patients and lymph node biopsies in 17 patients (Table 1). A complete set of both PET-CT data and lymphoid tissue was collected in 14 patients. Apart from mild infusion-related reactions (e.g. headache, transient drop in blood pressure) to the therapeutic dose of rituximab, no serious side effects of rituximab infusions were observed. ^89^Zr-rituximab was injected without any side effects.

**Table 1:** Baseline patient demographics, clinical and functional characteristics

	Responders (*n* = 13)	Non-responders (*n* = 7)	*P* value
Female, number (%)	12 (92)	6 (86)	0.589
Age, years	51.8 ± 13.3	54.4 ± 5.5	0.621
Length, cm	165.8 ± 8.7	166.6 ± 6.7	0.835
Weight, kg	71.2 (62.5–89.7)	70.0 (57.0–91.0)	0.643
IgM RF positivity, number (%)	10 (77)	5 (71)	0.594
RF titer, IU/mL	10.0 (8.4–84.5)	25.0 (0.0–176.0)	0.765
Anti-CCP positivity, number (%)	9 (79)	5 (71)	0.664
aCCP titer, U/mL	27.0 (0.0–193.5)	82.0 (0.0–270)	0.757
Disease duration, years	8.0 (3.5–16.0)	9.0 (4.0–21.0)	0.757
Current smokers, number (%)	3 (23)	3 (43)	0.336
DAS28	5.6 (4.9–6.1)	5.0 (4.0–7.1)	0.643
Swollen joint count	11.0 (7.5–14.0)	11.0 (8.0–18.0)	0.938
Tender joint count	14.0 (6.5–16.0)	8.0 (0.0–20.0)	0.588
HAQ	1.4 (0.8–1.8)	1.1 (0.5–2.0)	0.938
VAS	60.0 (52.5–64.0)	70.0 (40.0–75.0)	0.485
CRP, mg/mL^a^	6.0 (2.8–18.0)	4.0 (2.5–32.0)	0.699
ESR, mm/h	21.0 (10.5–41.0)	12 (5.0–36.0)	0.485
DMARD use, number (%)	12 (92)	5 (71)	0.270
Prednisone, number (%)	6 (46)	2 (29)	0.642
Dosage in mg/day	5 (0.0–7.5)	7.5 (0.0–10.0)	0.438

Values are presented as mean ± SD or median (IQR). ^a^CRP lower detection limit is 2.5 mg/mL. *IgM RF* rheumatoid factor, *anti-CCP* anti-cyclic citrullinated peptide, *DAS28* disease activity score of 28 joints, *HAQ* health assessment questionnaire, *VAS* visual analogue scale for pain, *CRP* C-reactive protein, *ESR* erythrocyte sedimentation rate, *DMARD* disease-modifying anti-rheumatic drugs

Baseline characteristics were comparable and no significant differences were found between clinical and serological data in responders and non-responders.

### PET-CT analysis

#### Tracer joint uptake and clinical response

**Fig. 2:**
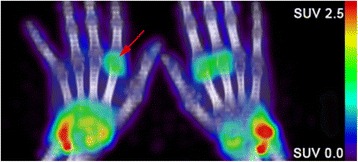
Example of a ^89^Zr-rituximab positron emission tomography (PET) image of the wrists/hands of a patient with rheumatoid arthritis who had multiple PET-positive joints. *Red arrow* represents one PET-positive joint (*green/yellow*). *SUV* standardized uptake values

In 18/20 patients there was clearly enhanced uptake in the joints of the wrists and hands, ranging from 1 to 20 joint(s) per patient (Fig. 2). Most (74%) of the PET-positive joints also had clinical signs of arthritis. Visual (dichotomous) interpretation of PET positivity in the joints did not distinguish between clinical responders and non-responders. Two patients who did not have clear visual uptake in the peripheral joints were both (moderate) responders.

Assessment in a subgroup of six patients with PET data available at 3 and 6 days p.i., showed stable standardized uptake values (SUV) in the hand joints over time. In contrast, a significant decrease of 25 ± 10.8% in ^89^Zr-rituximab was observed in the image-derived blood pool over time, resulting in significantly increased joint-to-blood ratios from a ratio of 0.4 to a ratio of 0.5 (*p* = 0.04). However, because of better count statistics and qualitative images at day 3, the results presented subsequently apply to the 3-day p.i. data.

**Fig. 3:**
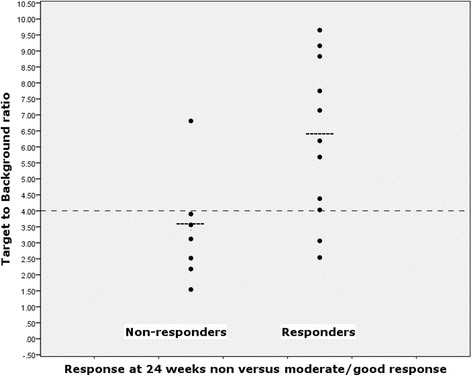
Target-to-background ratios of positron emission tomography (PET)-positive joints in relation to clinical response, in those patients who had at least one PET-positive joint on visual interpretation

Quantitative analysis of the hand joints of all 20 patients showed a trend of higher ^89^Zr-rituximab joint uptake in responders than in non-responders (SUV, *p* = 0.08; T/B ratio, *p* = 0.06). Moreover, among patients with at least one PET-positive joint after visual screening (*n* = 18), mean quantitative ^89^Zr-rituximab uptake in PET-positive hand joints per patient was significantly higher in responders vs. non-responders with respectively a mean SUV of 3.0 (2.5–3.5) vs. 1.9 (1.0–2.5) (*p* = 0.04). The majority of non-responders (6/7) had a mean SUV value below ≤2.5 in visual PET-positive joints, while the mean SUV values of responders was ≥2.5 in 9/11 patients. In line with the absolute quantitative joint uptake of ^89^Zr-rituximab, responders had a higher mean T/B ratio of PET-positive hand joints than non-responders 6.2 (4.0–8.8) vs. 3.1 (2.2–3.9) (*p* = 0.02), with a T/B ratio ≤4.0 for 6/7 non-responders (Fig. 3).

Interestingly, if quantitative analysis of the hand joints was limited to the PET-positive joint with the highest SUV, there were similar statistical differences in the SUV and T/B ratio between responders and non-responders. The significant differences between responders and non-responders were independent of serological status. Although exploratory, we further investigated the potential diagnostic values of our findings. Using a cutoff value of 4.0 for the T/B ratio, positive predictive value (PPV) and negative predictive value (NPV) for response in our cohort were respectively 90% (95% CI 55.5–99.8%) and 75% (95% CI 34.9–96.8%), at a sensitivity of 82% (95% CI 48.2–97.7%) and specificity of 86% (95% CI 42.1–99.6%) (Fig. 3).

### Whole body ^89^Zr-rituximab biodistribution and clinical response

**Fig. 4:**
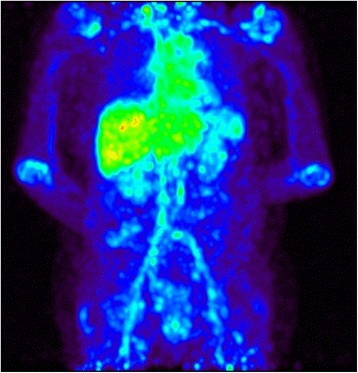
Maximum intensity projection whole-body ^89^Zr-rituximab positron emission tomography image, demonstrating the biodistribution in a patient with rheumatoid arthritis

**Fig. 5:**
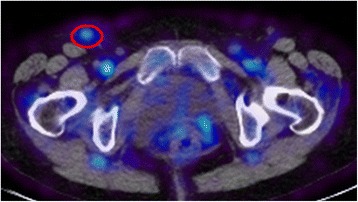
^89^Zr-rituximab positron emission tomography image-computer tomography image of a patient with rheumatoid arthritis, who had enhanced uptake in an inguinal lymph node (*red circle*)

Whole body PET-CT scans of all 20 patients showed uptake of ^89^Zr-rituximab in liver, spleen, kidneys, blood pool, and in large joints (e.g. shoulders) in 9 patients (Figs. 4 and 5). There were no significant differences in organ biodistribution between responders and non-responders (data not shown, see Additional file 3: Appendix C). Finally, quantitative PET measures in both body organs and detailed hand joints did not correlate with any clinical or laboratory baseline parameters, which are summarized in Table 1.

### PET imaging data of lymph nodes and histological evaluation

Lymph node biopsies were obtained in 13/20 patients and were compared to PET analysis. PET-positive lymph nodes were observed in 9/20 patients with RA and a lymph node biopsy was available in 6 of these patients. No association was found between visual PET positivity in lymph nodes and clinical response. Interestingly, however, quantitative ^89^Zr-rituximab uptake in the most clearly PET-positive lymph node (highest SUV per patient) was associated with CD22^+^ B-cell count in histological analysis of excised lymph nodes at baseline (*r* = 0.829; *p* = 0.04; *n* = 6). Although no associations were found between quantitative ^89^Zr-rituximab in lymph nodes on PET and CD22^+^ Bcell counts in excised lymph nodes at 4 weeks of rituximab treatment, Bcell counts in lymph nodes after 4 weeks treatment correlated negatively with baseline mean quantitative ^89^Zr-rituximab uptake in PET-positive hand joints (*r* = −0.6; *p* = 0.05).

## Discussion

Our study is the first that applied novel, non-invasive PET imaging of B cells by ^89^Zr-rituximab to investigate whether biodistribution of rituximab at baseline is related to clinical response to rituximab treatment. ^89^Zr-rituximab PET demonstrated clinically active joints, even partly in clinically silent joints, and there was a significant association between quantitative ^89^Zr-rituximab uptake in the hand joints and clinical response to rituximab treatment at 24 weeks. Moreover, by using a T/B cutoff value of 4.0 in PET-positive hand joints we found a potential positive predictive value of 90% for clinical response after 24 weeks rituximab treatment, while clinical and serological data were not distinctive. Apart from quantitative differences in joint uptake, there were no ^89^Zr-rituximab biodistribution differences in the lymph nodes or internal organs between responders and non-responders.

Specific targeting of B cells by ^89^Zr-rituximab was supported by positive associations between quantitative lymph node PET data and baseline CD22^+^ cell count (as a surrogate marker for B cells) in histological lymph node analysis, and by positive associations between post-treatment CD22^+^ B cells in lymph nodes and quantitative ^89^Zr-rituximab uptake on PET in the hand joint. These findings are in line with results from our colleagues, Jauw et al*.* who found that tumor uptake of ^89^Zr-rituximab correlated positively with CD20 expression in tumor biopsies in patients with diffuse, large Bcell lymphoma [22]. Other observations in this study also underlined the specificity of targeting B cells. Besides retention of ^89^Zr-rituximab in arthritic joints over time (while cleared from blood), specificity of uptake of ^89^Zr-rituximab in arthritic joints was also supported by our finding of significantly higher ^89^Zr-rituximab uptake in the joints of responders vs. non-responders, despite identical levels of disease activity at baseline. The lack of association between lymph node uptake on PET and clinical response may have been caused by the limited spatial resolution of PET of 4 mm, thus, positive lymph nodes may have been missed by PET.

Potentially, ^89^Zr-rituximab administration following the therapeutic dose of rituximab could result in competition of CD20 binding in the target, although it was administered within one hour. Nevertheless we chose this design to show the biodistribution of rituximab as used in the therapeutic setting in daily clinical practice. The kinetics of antibody influx in inflammatory targets are rather slow (from hours up to several days) [11], but partial blockade of CD20 binding sites by unlabeled rituximab at the time of the labeled rituximab infusion cannot be excluded. If therapeutic doses negatively influenced binding of the tracer then we may even have underestimated ^89^Zr-rituximab uptake in the synovium and lymph nodes. On the other hand, targeting of ^89^Zr-rituximab in the joints and lymph nodes may also be influenced positively by infusion of the therapeutic dose of rituximab just prior to injection of labeled ^89^Zr-rituximab, as the spleen has been recognized as the “sink” for rituximab binding during the first passage in circulation [11, 13]. Therefore, after saturation of the spleen, more ^89^Zr-rituximab may have become available for other CD20 targets such as the arthritic joints. For future clinical mAb studies in RA, dose escalation studies could answer this question and help define the optimal study design.

The finding that PET imaging with a ^89^Zr-labeled therapeutic antibody is able to predict therapeutic response of the antibody is in line with recent findings by Gebhart et al*.* They showed that pre-treatment ^89^Zr-trastuzumab imaging in combination with early ^18^F-FDG PET response assessment after one cycle of trastuzumab was promising for the identification of non-responders after three cycles (PPV100% and NPV92%) in patients with breast cancer [23].

There were 2 out of 20 patients in our study who did not show any ^89^Zr-rituximab uptake in the joints despite having a clinical response. The explanations for this may be relatively low Bcell counts (these patients had approximately 1500 CD22^+^ cells/mm^2^ in the lymph nodes vs. approximately 3000 cells/mm^2^ in PET-positive patients) and/or general low inflammatory activity in the joints in these patients, even though they did not differ clinically from other (PET-positive) responders. Finally, PET scans may have been false negative in these two patients.

Apart from ^89^Zr-rituximab PET, the serological status (RF and/or anti-CCP) has previously also been indicated as a potential predictive biomarker of therapeutic response. A meta-analysis, analyzing four placebo-controlled, phase II or phase III clinical trials, indicates that seropositive patients respond better to rituximab than seronegative patients [24]. We could not confirm this in our cohort and this discrepancy can be due to the relatively small sample size of this study. Nevertheless, in this relatively small number of patients, the PET approach did reveal the predictive potential of ^89^Zr-rituximab PET imaging with discriminative value between responders and non-responders by demonstrating and quantifying the radiolabeled drug in arthritic joints. Actually, the level of ^89^Zr-rituximab uptake in the hand joints did not correlate with any clinical or laboratory parameter at baseline. None of the clinical or laboratory data collected at baseline in our study differed between responders and non-responders.

## Conclusions

In conclusion, Bcell imaging in joints by ^89^Zr-rituximab PET-CT showed a clear association with clinical response at 24 weeks in patients with RA. This technique has potential value to select potential responders before initiation of rituximab treatment. This finding should be validated in larger cohorts, also in relation to other potential predictive biomarkers, in particular the serological status. Potentially, non-invasive, whole body ^89^Zr-rituximab PET-CT also holds promise for stratification and monitoring of anti-Bcell therapies in other Bcell-driven autoimmune diseases, such as systemic lupus erythematosus and Sjögren’s disease.

## Additional files

Additional file 1: Appendix A. Technical PET-CT details. More extensive information and details about the PET-CT scanning procedure and analysis methods. (DOCX 17 kb)
Additional file 2: Appendix B. Technical details about lymph node biopsy analysis. More extensive information and details about the lymph node biopsy tissue handling and analysis (immunohistochemistry). (DOCX 18 kb)
Additional file 3: Appendix C. Quantitative tissue uptake of ^89^Zr-rituximab on whole body PET in responders vs. non-responders. An additional table with SUV of body organs like liver, spleen and vertebrae showing that there is no (significant) difference between responders and non-responders in organ uptake of ^89^Zr-rituximab. (DOCX 15 kb)

## Abbreviations

^18^F-FDG: fluorodeoxyglucose
anti-CCP: anti-cyclic citrullinated peptide
anti-TNF: anti-tumor necrosis factor-alpha
CI: confidence interval
CRP: C-reactive protein
CT: computer tomography
DAS28: disease activity score of 28 joints
DMARDS: disease-modifying anti-rheumatic drugs
ESR: erythrocyte sedimentation rate
EULAR: European League Against Rheumatism
GBq/mL: Giga Becquerel per milliliter
GMP: good manufacturing practice
HAQ: health assessment questionnaire
i.v.: intravenous
IHC: immunohistochemical
IQR: interquartile range
mAb: monoclonal antibody
MBq: mega Becquerel
MCP: metacarpophalangeal
Mg: milligram
NaCl: sodium chloride
NPV: negative predictive value
p.i.: post injection
PET: positron emission tomography
PIP: proximal interphalangeal
PPV: positive predictive value
RA: rheumatoid arthritis
RF: rheumatoid factor
SD: standard deviation
SUV: standardized uptake values
T/B: target-to-background
VOI(s): volume(s) of interest

## References

[1] Edwards JC, Szczepanski L, Szechinski J, Filipowicz-Sosnowska A, Emery P, Close DR (2004). Efficacy of B-cell-targeted therapy with rituximab in patients with rheumatoid arthritis. N Engl J Med.

[2] Higashida J, Wun T, Schmidt S, Naguwa SM, Tuscano JM (2005). Safety and efficacy of rituximab in patients with rheumatoid arthritis refractory to disease modifying antirheumatic drugs and anti-tumor necrosis factor-alpha treatment. J Rheumatol.

[3] Porter D, van Melckebeke J, Dale J, Messow CM, McConnachie A, Walker A (2016). Tumour necrosis factor inhibition versus rituximab for patients with rheumatoid arthritis who require biological treatment (ORBIT): an open-label, randomised controlled, non-inferiority, trial. Lancet.

[4] Emery P, Deodhar A, Rigby WF, Isaacs JD, Combe B, Racewicz AJ (2010). Efficacy and safety of different doses and retreatment of rituximab: a randomised, placebo-controlled trial in patients who are biological naive with active rheumatoid arthritis and an inadequate response to methotrexate (Study Evaluating Rituximab's Efficacy in MTX iNadequate rEsponders (SERENE)). Ann Rheum Dis.

[5] Tak PP, Rigby W, Rubbert-Roth A, Peterfy C, van Vollenhoven RF, Stohl W (2012). Sustained inhibition of progressive joint damage with rituximab plus methotrexate in early active rheumatoid arthritis: 2-year results from the randomised controlled trial IMAGE. Ann Rheum Dis.

[6] Jones T (1996). The role of positron emission tomography within the spectrum of medical imaging. Eur J Nucl Med.

[7] Gent YY, Ter Wee MM, Voskuyl AE, den Uyl D, Ahmadi N, Dowling C (2015). Subclinical synovitis detected by macrophage PET, but not MRI, is related to short-term flare of clinical disease activity in early RA patients: an exploratory study. Arthritis Res Ther.

[8] Gent YYJ, Voskuyl AE, Kloet RW, van Schaardenburg D, Hoekstra OS, Dijkmans BAC (2012). Macrophage positron emission tomography imaging as a biomarker for preclinical rheumatoid arthritis: findings of a prospective pilot study. Arthritis Rheum.

[9] Elzinga EH, van der Laken CJ, Comans EF, Boellaard R, Hoekstra OS, Dijkmans BA (2011). 18 F-FDG PET as a tool to predict the clinical outcome of infliximab treatment of rheumatoid arthritis: an explorative study. J Nucl Med.

[10] Roivainen A, Hautaniemi S, Mottonen T, Nuutila P, Oikonen V, Parkkola R (2013). Correlation of 18 F-FDG PET/CT assessments with disease activity and markers of inflammation in patients with early rheumatoid arthritis following the initiation of combination therapy with triple oral antirheumatic drugs. Eur J Nucl Med Mol Imaging.

[11] van Dongen GA, Huisman MC, Boellaard R, Harry HN, Windhorst AD, Visser GW (2015). 89Zr-immuno-PET for imaging of long circulating drugs and disease targets: why, how and when to be applied?. Q J Nucl Med Mol Imaging.

[12] Vosjan MJ, Perk LR, Visser GW, Budde M, Jurek P, Kiefer GE (2010). Conjugation and radiolabeling of monoclonal antibodies with zirconium-89 for PET imaging using the bifunctional chelate p-isothiocyanatobenzyl-desferrioxamine. Nat Protoc.

[13] Muylle K, Flamen P, Vugts DJ, Guiot T, Ghanem G, Meuleman N (2015). Tumour targeting and radiation dose of radioimmunotherapy with (90)Y-rituximab in CD20+ B-cell lymphoma as predicted by (89)Zr-rituximab immuno-PET: impact of preloading with unlabelled rituximab. Eur J Nucl Med Mol Imaging.

[14] Bugatti S, Vitolo B, Caporali R, Montecucco C, Manzo A. B cells in rheumatoid arthritis: from pathogenic players to disease biomarkers. Biomed Res Int. 2014;2014:1–14. 10.1155/2014/681678 PMC4022166 24877127

[15] Moller B, Aeberli D, Eggli S, Fuhrer M, Vajtai I, Vogelin E (2009). Class-switched B cells display response to therapeutic B-cell depletion in rheumatoid arthritis. Arthritis Res Ther.

[16] Thurlings RM, Vos K, Wijbrandts CA, Zwinderman AH, Gerlag DM, Tak PP (2008). Synovial tissue response to rituximab: mechanism of action and identification of biomarkers of response. Ann Rheum Dis.

[17] Verel I, Visser GW, Boellaard R, Stigter-van WM, Snow GB, van Dongen GA (2003). 89Zr immuno-PET: comprehensive procedures for the production of 89Zr-labeled monoclonal antibodies. J Nucl Med.

[18] Makris NE, Boellaard R, Visser EP, de Jong JR, Vanderlinden B, Wierts R (2014). Multicenter harmonization of 89Zr PET/CT performance. J Nucl Med.

[19] de Hair MJ, Zijlstra IA, Boumans MJ, van de Sande MG, Maas M, Gerlag DM (2012). Hunting for the pathogenesis of rheumatoid arthritis: core-needle biopsy of inguinal lymph nodes as a new research tool. Ann Rheum Dis.

[20] Haringman JJ, Vinkenoog M, Gerlag DM, Smeets TJ, Zwinderman AH, Tak PP (2005). Reliability of computerized image analysis for the evaluation of serial synovial biopsies in randomized controlled trials in rheumatoid arthritis. Arthritis Res Ther.

[21] van Gestel AM, Prevoo ML, van 't Hof MA, van Rijswijk MH, van de Putte LB, van Riel PL. Development and validation of the European League Against Rheumatism response criteria for rheumatoid arthritis. Comparison with the preliminary American College of Rheumatology and the World Health Organization/International League Against Rheumatism Criteria. Arthritis Rheum. 1996;39(1):34–40 10.1002/art.1780390105 8546736

[22] Jauw YWS, Huisman MC, De Jong D, Vugts DJ, Zweegman S, Van Dongen GAMS, et al. 89Zr-labeled-rituximab PET as an imaging biomarker to assess CD20 targeting: a pilot study in patients with relapsed/refractory diffuse large B cell lymphoma. [Abstract] 6th international workshop on PET in lymphoma, Palais de l'Europe, Menton (France); 2016.

[23] Gebhart G, Lamberts LE, Wimana Z, Garcia C, Emonts P, Ameye L (2016). Molecular imaging as a tool to investigate heterogeneity of advanced HER2-positive breast cancer and to predict patient outcome under trastuzumab emtansine (T-DM1): the ZEPHIR trial. Ann Oncol.

[24] Isaacs JD, Cohen SB, Emery P, Tak PP, Wang J, Lei G (2013). Effect of baseline rheumatoid factor and anticitrullinated peptide antibody serotype on rituximab clinical response: a meta-analysis. Ann Rheum Dis.

