# Supplementary material for: B-cell imaging with zirconium-89 labelled rituximab PET-CT at baseline is associated with therapeutic response 24 weeks after initiation of rituximab treatment in rheumatoid arthritis patients
Source: Arthritis Res Ther. 2016 Nov 18;18:266. doi: 10.1186/s13075-016-1166-z (PMC5116204; doi:10.1186/s13075-016-1166-z)
Supplement: Additional file 1: — Appendix A. Technical PET-CT details. More extensive information and details about the PET-CT scanning procedure and analysis methods. (DOCX 17 kb) [file 13075_2016_1166_MOESM1_ESM.docx]

Appendix A: Technical PET-CT details

PET-CT scanning

Scans were obtained with PET-CT types Philips Medical Systems Gemini TF (Cleveland, Tennessee, USA) or Ingenuity TF(Cleveland, Ohio, USA). Patients were positioned supine, feet first. After four patients an interim analysis was performed for protocol optimization. Whole body scans of 10 minutes per field of view (FOV) from shoulders to upper legs were acquired. In addition, detailed static emission scans of 15 minutes, a single bed position of hands/wrists were obtained. Patients were positioned supine, head first, with their hands in a special vaccuum pouch for stabilisation of the hands. All PET scans were preceded by a low dose 35 mAs CT scan which was used for attenuation correction and localization of PET signal. The maximum total scan time was approximately 60-75 minutes per patient.
 PET data were normalized and corrected for attenuation, decay and scatter. All scans were reconstructed according to international guidelines (1). These reconstructed images were also used for volume of interest (VOI) definition.

PET imaging Analysis

Whole body PET-CT images were qualitatively interpreted by one experienced nuclear medicine specialist (OSH) and were visually interpreted for biodistribution pattern and extra-articular uptake (e.g. lymph nodes). Detailed images of wrists/hands, were subsequently interpreted by two independent readers (OSH and SBR) for PET positivity (dichotomous) of 22 hand/wrist joints, excluding the distal interphalangeal (DIP) joints. A third reader (JvdL) was consulted in case of discrepancies. All readers were blinded for clinical data.

For quantitative analysis, VOIs were drawn using analyzing software developed in-house (1) with the covering low dose CT as anatomical reference. To analyze 89Zr-RTX biodistribution in the body, VOIs were drawn on the lungs, liver, spleen, kidneys, aortic arch (representing blood pool activity) (fixed size VOI of 1.6 cc in the aortic arc), bone marrow (fixed size VOI of 1 cc in resp. L3,L4 and L5) and lymph nodes (using thresholds versus local background), using in-house biodistribution standard operating procedures. Average standard uptake values (SUVaverage), defined as mean uptake in the VOI divided by injected dose and patient weight, were calculated.

For comparison of quantitative joint uptake in wrist/hands on PET with clinical follow-up, VOIs were drawn on top of visually marked PET positive joints using fixed lower level thresholds considering local background uptake values. As advised by Makris et al., tracer uptake in targets is presented as SUV 3Dpeak (1). A 3Dpeak value is based on a sphere of 1.2cm in diameter (1cc) within the VOI, with the highest average uptake (1). In addition, spherical VOIs of 0.5cc in preferably the second metacarpal bone were used as background to calculate local target-to-background (T/B) ratios. Because this VOI is <1cc, SUVpeak is not representative and hence background is presented as SUVaverage.
 To be able to compare quantitative uptake of all joints (clinically active versus clinically inactive joints and PET positive versus negative joints) we applied fixed sized VOIs. Such VOIs were drawn on wrists (~60 cc), metacarpophalangeal (MCP) joints 1-5 (~8 cc) and proximal interphalangeal (PIP) joints 1-5 (~2 cc) centered in the middle of the joint.

Reference List

(1) Makris NE, Boellaard R, Visser EP, de Jong JR, Vanderlinden B, Wierts R et al. Multicenter harmonization of 89Zr PET/CT performance. J Nucl Med 2014; 55(2):264-7.
