# Supplementary material for: B-cell imaging with zirconium-89 labelled rituximab PET-CT at baseline is associated with therapeutic response 24 weeks after initiation of rituximab treatment in rheumatoid arthritis patients
Source: Arthritis Res Ther. 2016 Nov 18;18:266. doi: 10.1186/s13075-016-1166-z (PMC5116204; doi:10.1186/s13075-016-1166-z)
Supplement: Additional file 2: — Appendix B. Technical details about lymph node biopsy analysis. More extensive information and details about the lymph node biopsy tissue handling and analysis (immunohistochemistry). (DOCX 18 kb) [file 13075_2016_1166_MOESM2_ESM.docx]

Appendix B: Technical details lymph node biopsy analysis.

Immunohistochemistry

Lymph node sections of 5 μm were mounted on StarFrost adhesive glass slides (Knittelgläser, Braunschweig, Germany) and stored at −80°C. Sections were stained using mouse monoclonal antibodies against B-cells (anti-CD22, clone RFB4; Millipore, Amsterdam, the Netherlands) and T-cells (anti-CD3, clone SK7 (Becton Dickinson, Breda, the Netherlands). For detection of CD22 B-cells and CD3 T-cells a 2-step immunoperoxidase method with a secondary polymer–horseradish peroxidase–conjugated anti-mouse antibody (EnVision+ System; Dako) was used. In contrast, plasma cells (anti-CD138, clone MI15; Dako, Heverlee, Belgium) were detected by a 3-step immunoperoxidase method as described previously(1). In addition, irrelevant isotype-matched immunoglobulins were applied as a negative control. Staining was analysed by digital image analysis using a Syndia algorithm on a Qwin-based analysis system (Leica, Cambridge, UK) as described previously (2). The number of positive cells was calculated as the number of positive cells per square millimetre of stained tissue.

Reference List

(1) Tak PP, van der Lubbe PA, Cauli A, Daha MR, Smeets TJ, Kluin PM et al. Reduction of synovial inflammation after anti-CD4 monoclonal antibody treatment in early rheumatoid arthritis. Arthritis Rheum 1995; 38(10):1457-65.

(2) Haringman JJ, Vinkenoog M, Gerlag DM, Smeets TJ, Zwinderman AH, Tak PP. Reliability of computerized image analysis for the evaluation of serial synovial biopsies in randomized controlled trials in rheumatoid arthritis. Arthritis Res Ther 2005; 7(4):R862-R867.
