# Supplementary material for: B-cell imaging with zirconium-89 labelled rituximab PET-CT at baseline is associated with therapeutic response 24 weeks after initiation of rituximab treatment in rheumatoid arthritis patients
Source: Arthritis Res Ther. 2016 Nov 18;18:266. doi: 10.1186/s13075-016-1166-z (PMC5116204; doi:10.1186/s13075-016-1166-z)
Supplement: Additional file 3: — Appendix C. Quantitative tissue uptake of 89Zr-rituximab on whole body PET in responders vs. non-responders. An additional table with SUV of body organs like liver, spleen and vertebrae showing that there is no (significant) difference between responders and non-responders in organ uptake of 89Zr-rituximab. (DOCX 15 kb) [file 13075_2016_1166_MOESM3_ESM.docx]

Appendix C

Quantitative tissue uptake of ^89^Zr-rituximab on whole body PET in responders versus non-responders.

| Baseline tissue uptake (SUVaverage) | Responders (n=13) | Non-responders  (n=7) | P-value |
| --- | --- | --- | --- |
| Bloodpool (Aortic Arch) | 5.8 [5.1-6.7] | 5.6 [5.1-8.4] | 0.758 |
| Liver | 5.4 [5.2-6.1] | 5.6 [4.7-7.4] | 0.758 |
| Spleen | 3.5 [3.2-4.0] | 3.3 [2.9-3.8] | 0.351 |
| Kidneys | 3.9 [3.5-4.2] | 3.5 [3.1-4.7] | 0.758 |
| Lungs | 2.0 [1.8-2.3] | 1.9[1.5-2.6] | 0.918 |
| Vertebrae | 2.0 [1.7-2.7] | 2.4 [1.9-2.7] | 0.536 |
| Lymph nodes | 1.0 [0.6-2.1] | 1.6 [1.2-2.4] | 0.343 |
